# A novel human artery model to assess the magnetic accumulation of SPIONs under flow conditions

**DOI:** 10.1038/srep42314

**Published:** 2017-02-08

**Authors:** Agata Janikowska, Jasmin Matuszak, Stefan Lyer, Eveline Schreiber, Harald Unterweger, Jan Zaloga, Jürgen Groll, Christoph Alexiou, Iwona Cicha

**Affiliations:** 1 Section of Experimental Oncology und Nanomedicine (SEON), ENT-Department, University Hospital Erlangen, Erlangen, Germany; 2 Department of Functional Materials in Medicine and Dentistry, University Hospital Würzburg, Würzburg, Germany

## Abstract

Magnetic targeting utilises the properties of superparamagnetic iron oxide nanoparticles (SPIONs) to accumulate particles in specified vasculature regions under an external magnetic field. As the behaviour of circulating particles varies depending on nanoparticle characteristics, magnetic field strength and flow dynamics, we established an improved *ex vivo* model in order to estimate the magnetic capture of SPIONs in physiological-like settings. We describe here a new, easy to handle *ex vivo* model of human umbilical artery. Using this model, the magnetic targeting of different types of SPIONs under various external magnetic field gradients and flow conditions was investigated by atomic emission spectroscopy and histology. Among tested particles, SPION-1 with lauric acid shell had the largest capacity to accumulate at the specific artery segment. SPION-2 (lauric acid/albumin-coated) were also successfully targeted, although the observed peak in the iron content under the tip of the magnet was smaller than for SPION-1. In contrast, we did not achieve magnetic accumulation of dextran-coated SPION-3. Taken together, the umbilical artery model constitutes a time- and cost-efficient, 3R-compliant tool to assess magnetic targeting of SPIONs under flow. Our results further imply the possibility of an efficient *in vivo* targeting of certain types of SPIONs to superficial arteries.

Among the wide variety of nanoparticle systems which are being studied for the purpose of medical applications, magnetic nanoparticles represent a versatile platform that can be potentially utilized both as a diagnostic (contrast) agent and as a drug delivery system. The latter application can be greatly improved by active targeting to allow a better control of nanoparticle biodistribution and to enhance their therapeutic efficacy. For magnetic nanoparticles, a promising strategy of drug delivery, which results in increased drug payloads in the target tissue, at the same time reducing their systemic dose and toxicity, is based on so-called magnetic drug targeting (MDT). In this approach, conjugation of superparamagnetic iron oxide nanoparticles (SPIONs) with drugs in combination with an external magnetic field is used to target the particles to the diseased vasculature regions as demonstrated by the studies in a rabbit model of cancer^1^,^2^,^3^, a mouse model of thrombosis^4^, a mouse model of cardiac ischemia^5^ and several mouse models of cancer^6^,^7^,^8^. The existing studies mostly utilized the phenomenon of an enhanced permeability of the microvessel endothelium in cancer and inflammatory diseases, which facilitates the extravasation of nano-sized particles^9^.

Magnetic capture under flow conditions characteristic for larger vessels occurs when the force exerted on the particles by the magnetic field overcomes the particle hydrodynamic (drag) force. Hence, the behaviour of magnetic particles in circulation may vary greatly depending on the nanoparticle characteristics, the magnetic field gradients and the flow dynamics. In particular, the effect of particle-particle interactions under the influence of magnetic field gradients is a very complex matter^10^ and the extent its contribution to MDT is not yet fully understood. In physiological conditions, arterial wall is constantly exposed to shear stress induced by the flow of blood and its viscosity, and the patterns of shear stress affect the behaviour of the blood-borne cells and particles^11^. Thus far, the experimental attempts to magnetically target medium and large vessels have been very scarce^12^,^13^,^14^. It is therefore important to investigate the possibility of accumulation of magnetic particles under physiologic-like flow conditions in the experimental models prior to the *in vivo* MDT application.

In our present report, we describe the development of an *ex vivo* model for investigations of magnetic accumulation of SPIONs based on the use of human umbilical cord arteries. Normal human umbilical cords contain 2 arteries and are usually disposed of as post-partum waste. The advantage of these arteries is their size (similar to human coronary arteries^15^) and the lack of any branches. In order to control the vessel diameter and distensibility, arteries are embedded in a supporting matrix. This new model, allowing improved standardisation, was employed here to investigate the efficacy of magnetic targeting, utilizing 3 types of SPIONs with different physicochemical characteristics. Furthermore, the effects of varying external magnetic field parameters and of the variation of SPION circulation time and flow conditions on the magnetic capture efficacy were analysed.

## Results

### Umbilical artery characterisation

The umbilical arteries used in this study had diameter of 2.15 ± 0.09 mm (mean ± SEM; median: 2.11 mm; range 1.32–2.98 mm) as measured in n = 18 histological specimens. The arteries were isolated from the umbilical cords between day 1 and day 4 post-partum. Only the intact artery fragments were used in order to avoid the leakage of SPIONs and unspecific accumulation in the artery wall. The example images of vessel morphology analysed by histological staining are shown in Suppl. Fig. 1. The phenotypic features and viability of human umbilical artery endothelial cells (HUAECs) isolated from the characterised arteries are summarized in Suppl. Figs 2 and 3. The arteries used in the present study were routinely isolated from the respective cords on the same day as human umbilical vein endothelial cells (HUVECs), which provided an independent control of the presence of viable cells in the umbilical vessels.

### *Ex vivo* human umbilical artery model

**Figure 1:**
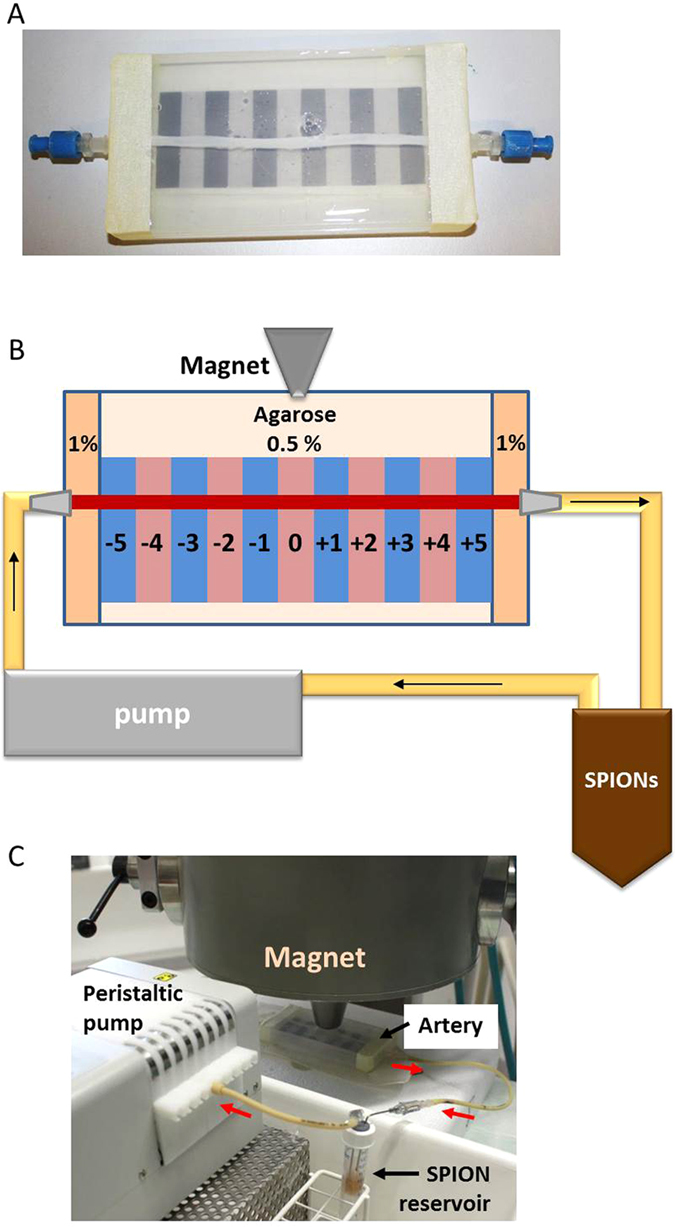
Umbilical artery model. (**A**) Umbilical artery embedded in agarose gel; (**B**) Schematic presentation of the experimental set-up for magnetic targeting; (**C**) Example image showing the electromagnet, the artery, SPION reservoir, and peristaltic pump. Red arrows indicate the flow direction.

To provide an *in vivo*-like mechanical support and control over the artery diameter, an entirely new set-up was established and optimised as described in detail in Methods. Artery fragments (13 cm long, see Fig. 1), rinsed multiple times with Ringer solution and filled with endothelial cell culture medium to prevent the lumen collapse, were embedded in agarose gels of different densities. After gel solidification, the artery fragments were perfused with medium at the flow rate of 4.8 mL/min, corresponding to the shear stress of 5 dyne/cm^2^, which is similar as occurring in medium-sized human arteries. Among the tested gel densities, the concentration of 0.5% agarose was found to provide good vessel support during the experiment, but did not secure the ends attached to the connectors to prevent minor leakage due to the pressure difference resulting from the flow of medium. Therefore, external 1 cm segments on both ends of the artery (in-flow and out-flow segments) were mounted in more condensed 1% agarose gel. These conditions were selected as the final set-up, which was subsequently used in the experiments with SPIONs.

### Magnetic capture efficacy of different types of SPIONs

In our present study, magnetic accumulation of three types of SPIONs with different coatings and physicochemical properties was evaluated: SPION-1 with 126 nm hydrodynamic diameter and ζ-potential of −34.6 mV, SPION-2 with 79 nm hydrodynamic diameter and ζ-potential of −37.3 mV, and SPION-3 with 78 nm hydrodynamic diameter and ζ-potential of +0.1 mV (see Table 1 and Methods). The selected concentration of circulating SPIONs (30 μg/mL) was comparable to the maximal systemic dose of iron oxide-based contrast agent ferumoxtran (2.6 mg/kg body weight, corresponding to 33 μg/mL blood in humans).

**Figure 2:**
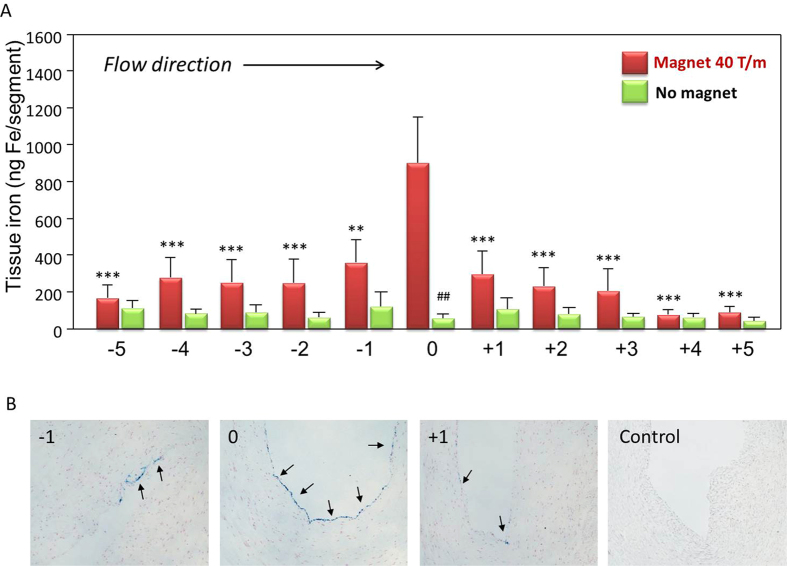
Magnetic targeting of circulating SPION-1. (**A**) SPION-1 suspension at 30 μg Fe/mL was circulated for 30 min under external magnetic field gradient positioned at segment “0” (red bars; the magnetic field gradient at the centre of artery: 40 T/m) or without magnetic force (green bars). Shown is iron concentration of respective segments (mean values ± SEM of n = 5 experiments). ***P < 0.001; **P < 0.01 vs region “0” under the tip of the magnet; ^##^P < 0.01 vs corresponding targeted region. (**B**) Representative images from n = 3 experiments corresponding to the artery segments −1, 0 and +1. Iron accumulation, visualised with Prussian blue staining, is highlighted with arrows.

The considerable differences in the physicochemical properties of SPIONs resulted in dramatic differences of their magnetic accumulation under flow conditions, as also shown in our previous *in vitro* studies^16^. Out of the three types of SPIONs tested in the *ex vivo* artery model, SPION-1 had by far the largest capacity to accumulate at the artery segment placed directly under the tip of the magnet, corresponding to the magnetic field gradient of about 40 T/m at the artery centre (Fig. 2A). As compared with untargeted samples, the magnetic targeting resulted in >10-fold increase in SPION-1 accumulation in the arterial segment localised directly under the tip of the magnet. The quantitative results of the iron content analysis obtained with atomic emission spectroscopy (AES) were subsequently confirmed qualitatively using histological staining. Figure 2B shows the artery segments at position 0 (directly under the tip of the magnet) and the positions −1 and +1. Nanoparticle accumulation reflected by blue staining shows clear differences depending on the distance from the “0” position.

**Figure 3:**
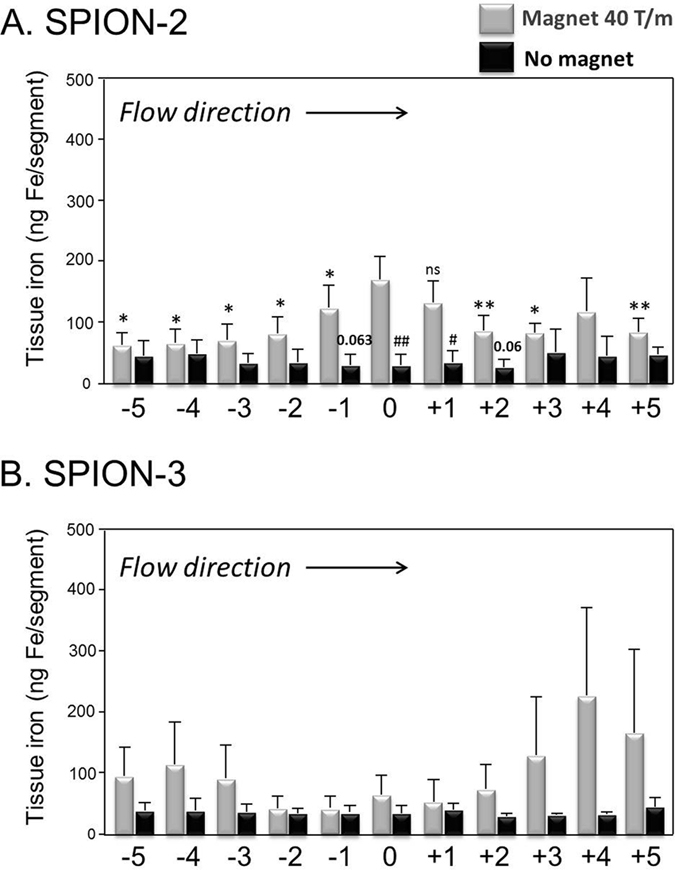
Magnetic targeting of circulating SPION-2 and SPION-3. (**A**) SPION-2 and (**B**) SPION-3 suspensions at 30 μg Fe/mL were circulated in the model for 30 min under external magnetic force positioned at segment “0” (grey bars; the magnetic field gradient at the centre of artery: 40 T/m) or without magnetic force (black bars). Shown is iron concentration of respective segments (mean values ± SEM of n = 5 experiments). **P < 0.01, *P < 0.05, ns – two-tailed P value of 0.097 vs region “0”; ^##^P < 0.01, ^#^P < 0.05 vs corresponding targeted region.

For circulating SPION-2, the observed peak in the iron content under the tip of the magnet was smaller than for SPION-1, but the difference in particle accumulation between (a) the targeted region versus more distant regions of the same artery and (b) magnetically targeted and un-targeted samples within regions 0 to +1 remained strongly pronounced and statistically significant (Fig. 3A). In contrast, no accumulation of SPION-3 was achieved (Fig. 3B). There were no significant differences in tissue iron content between magnetically targeted and un-targeted artery samples. At the inflow and outflow regions of the model, peaks in tissue iron accumulation were observed, which were most pronounced at +4 and +5 segments. As shown in the Fig. 3B, this accumulation of SPION-3 at the model outflow bears a very high error value, which resulted from the fact that such strong effect was observed in only 1 of the 5 samples that were quantified by AES.

**Figure 4:**
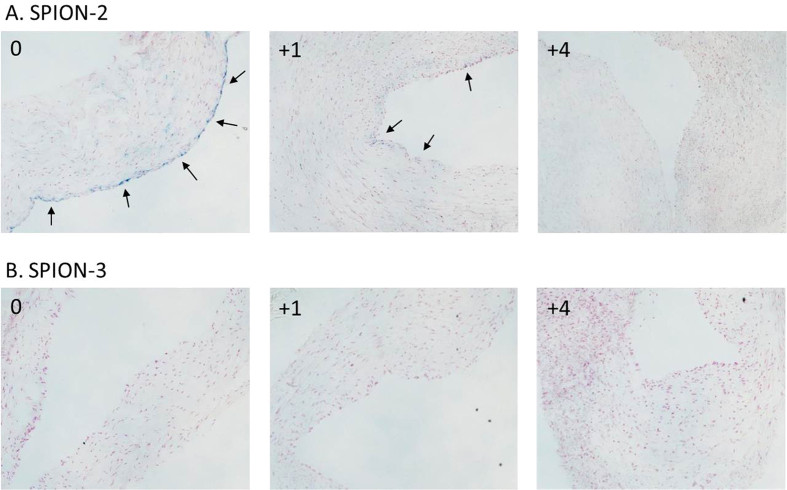
Histological evaluation of magnetic targeting. Representative images from n = 3 experiments corresponding to the artery segments 0, +1 and +4 are shown for (**A**) SPION-2 and (**B**) SPION-3. Iron accumulation, visualized with Prussian blue staining, is highlighted with arrows. No accumulation was detectable by histology in the analysed SPION-3 samples.

The results of the histological analyses in samples with circulating SPION-2 and SPION-3 are visualised in Fig. 4, showing the artery segments at position 0 (directly under the tip of the magnet) and the positions +1 and +4. Nanoparticle accumulation reflected by blue staining shows clear differences depending on the SPION type. It must be noted that the segments analysed histologically for the presence of SPION-3 (n = 3 independent experiments) were negative, independent of the region.

### Effects of magnetic field parameters on capture efficacy

**Figure 5:**
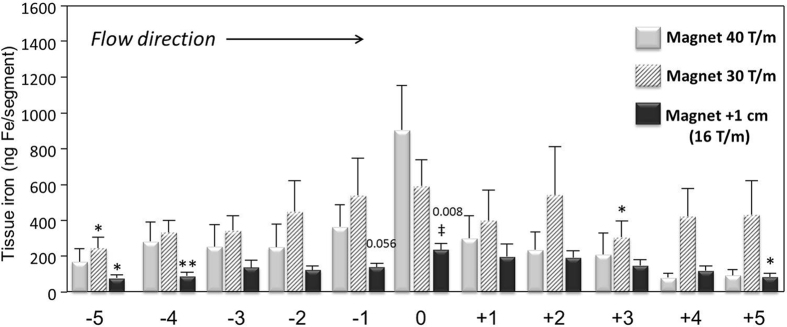
Effect of magnetic field parameters on magnetic targeting. SPION-1 suspension at 30 μg Fe/mL was circulated in the model for 30 min. The estimated magnetic field gradient at the centre of artery lumen was 40 T/m (grey bars) or 30 T/m (striped bars). In further experiments, tip of the magnet set to 72 T/m was positioned at 1 cm distance from agarose gel surface (total distance from the artery: 15 mm; 16 T/m at the centre of artery lumen; black bars). Shown is the iron concentration of respective segments (mean values ± SEM of n = 5 experiments). **P < 0.01, *P < 0.05 vs respective region “0”; ^‡^P < 0.05 vs region “0” at 40 T/m. The P values in “+1 cm” samples versus corresponding regions at 30 T/m are indicated.

To address the effect of magnetic field parameters on SPION accumulation, a series of experiments was performed with (a) reduced magnetic field gradient and (b) increased distance from the tip of the magnet to the artery. In those experiments, SPION-1 were applied, as these particles showed the highest iron peak under the magnetic pole shoe and therefore, we expected to detect the resulting changes in their accumulation with higher sensitivity. The results of these experiments are summarised in Fig. 5. A reduction from 72 to 36 T/m at the tip of the magnet caused the magnetic field gradient decrease from 40 to 30 T/m at the centre of artery lumen. In comparison to 40 T/m, this reduced magnetic field gradient resulted in the iron peak decrease, in parallel with slightly increased accumulation in segments −5 to −1 and +1 to +5, which led to an overall flattened distribution curve along the artery.

In the subsequent experiments, magnetic field gradient was set to 72 T/m at the magnetic pole shoe, but the tip of the magnet was positioned at 1 cm distance from the agarose gel surface, i.e. at total distance of about 15 mm from the artery wall. Since the magnetic field gradient decreases exponentially with an increasing distance, it was reduced to about 16 T/m at the artery lumen centre in those conditions, and a large effect of the increased distance on the capture efficacy was expected. As shown in Fig. 5 (black bars), under these conditions, the accumulation of SPION-1 under the tip of the magnet, as quantified by tissue iron content measurement, was reduced by about 75% (P < 0.05).

Concerning the potential *in vivo* applications, these data suggest that targeting with external magnet should allow SPION accumulation in the superficial arteries, but can prove difficult in the case of arteries localised in the deeper body regions. Both the broader particle distribution at the decreased magnetic field strength and the clear dependency of MDT efficacy on the distance from the tip of the magnet indicate that a certain threshold gradient exists that must be present to overcome the hydrodynamic forces of the flowing fluid in order to effectively accumulate the magnetic nanoparticles at the arterial wall.

### Effects of the flow time and rate on magnetic accumulation of circulating SPIONs

**Figure 6:**
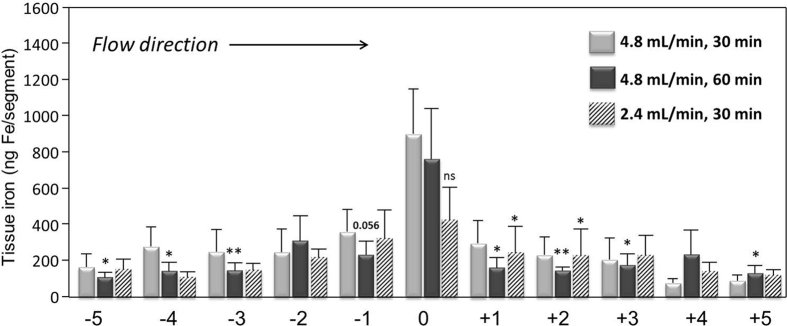
Effects of time and flow rate on magnetic targeting of circulating SPIONs. SPION-1 suspension at 30 μg Fe/mL was circulated for 30 min (grey bars) or 60 min (black bars). The magnetic field gradient at the centre of artery was 40 T/m. In further experiments, flow rate was reduced to 2.4 mL/min (striped bars). Shown is the iron concentration of respective segments (mean values ± SEM of n = 5 experiments). **P < 0.01, *P < 0.05 vs respective region “0”; ns, two-tailed P value = 0.16 vs region “0” at flow rate of 4.8 mL/min.

In further experiments, the effects of circulation time and flow rate were evaluated. For this purpose, SPION-1 were circulated in the model under the external magnetic force for 60 min (black bars). As shown in Fig. 6, this prolonged circulation time had no major effect on the accumulation of circulating SPION-1, which may indicate that the magnetic field gradient application for 30 min is sufficient to attract these nanoparticles.

Concerning the flow rate, about 50% decrease in SPION-1 accumulation was observed, when the flow rate was reduced from 4.8 to 2.4 mL/min (Fig. 6, striped bars). This was an unexpected result, which however can be explained by a strong sedimentation of SPION-1 observed at this flow rate. Based on this observation, it is plausible that the streamlines of flowing SPION-1 were localized near the bottom of the artery lumen, which increased the effective distance for magnetic field gradient to about 8–10 mm, leading to the observed tendency for decreased particle accumulation under the tip of the magnet.

## Discussion

The efficacy of magnetic drug targeting, both in terms of the amounts of delivered drug and the therapeutic outcome has been demonstrated in several studies on tumour-bearing rabbits treated intra-arterially with mitoxantrone-loaded SPIONs^1^,^2^, as well in the mouse models of cancer upon intravenous application of doxorubicin-loaded nanoparticles (Chao *et al*.^6^, Elbialy *et al*.^7^, Yu *et al*.^8^). Magnetic targeting was furthermore effective in a rat model of myocardial infarction reported by Zhang *et al*.^5^, where the externally-controlled magnetic nanobeads conjugated to adenoviral vectors-encoded human VEGF gene were administered intravenously. It must be noted, however, that the above-mentioned *in vivo* studies applied the external magnetic field gradient to the microvasculature regions characterized by relatively slow flow and in the disease conditions, where enhanced capillary leakage and retention effects strongly support the accumulation of SPIONs.

In the arteries, characterised by larger diameter and increased shear stress (varying between 3–7 dyne/cm^2^ in the peripheral arteries, and 10–15 dyne/cm^2^ in the central arteries^17^), the hemodynamic forces are predicted to critically affect the particle behaviour and internalisation. Theoretically, the targeting efficiency is expected to grow with increased magnetic forces, decreased flow rate and reduced vessel diameter^18^. Nevertheless, the *in silico* mathematical simulations based on the models of tubular vessels predict that magnetic accumulation of nanoparticles against the hydrodynamic drag force is not possible^19^. These models, however, often ignore dipole-dipole interactions between the magnetized particles. Recent simulations and experiments suggest that aggregates which are formed by interaction of SPIONs’ magnetic dipoles greatly contribute to magnetic attraction of SPIONs. Electrostatic or steric repulsion due to the surface chemistry also plays an important role^10^. The contribution of *in situ-*formed reversible aggregates during high gradient magnetic separation of magnetic nanoparticles has been described earlier by Moeser *et al*.^20^. In support of their hypothesis, a successful accumulation of circulating SPIONs under external magnets has been demonstrated in several *in vitro* models of straight^21^,^22^ and bifurcating channels^16^,^23^,^24^. Furthermore, our previous experimental *ex vivo* work in bovine arteries^14^,^25^ showed that accumulation of flowing SPIONs in the arterial wall is achievable under the guidance of a sufficiently strong external magnet, as confirmed by histology, microCT and magnetorelaxometry^14^,^26^. Despite these promising initial results, bovine carotid artery model was suboptimal due to the time-consuming procedures of isolation and closing the multiple branches. To answer the need for an easy to handle and reliable basic research model system for MDT investigations under arterial flow conditions, we therefore developed a new *ex vivo* model based on the branch-free human umbilical cord arteries. In this model, the magnetic capture efficacy of 3 types of SPIONs was investigated. By using particles differing in multiple physicochemical properties e.g. core sizes, stabilisation mechanisms and coatings, we showed that testing the magnetic accumulation of the circulating SPIONs *ex vivo* can provide critical information to predict their behaviour and the capture efficacy *in vivo*. As demonstrated here, SPION-1 had by far the largest capacity to accumulate at the artery segment exposed to strongest magnetic field gradient. Nanoparticles with a ζ-potential above (+/−) 30 mV are usually considered as colloidally stable, as the surface charge prevents their aggregation. Steric repulsion, such as the hindrance provided by a coating of the nanoparticle surface (e.g. SPION-3 with dextran shell), can also provide high colloidal stability despite a nearly neutral ζ-potential^27^. Comparing the formulations used here (SPION-1 with lauric acid shell, SPION-2 with lauric acid/BSA shell and SPION-3 with dextran shell), all types of SPIONs had good colloidal stability in water. However, in the case of SPION-1, a strong tendency to sediment and cluster was observed in cell culture media. It is therefore plausible that, in accordance with the findings of Moeser *et al*.^20^, this enhanced tendency to form aggregates results in an increased magnetic targeting of SPION-1 clusters under arterial flow conditions. Despite the fact that these particles constitute a very good model nanosystem for evaluation of various parameters influencing the magnetic capture under flow conditions, SPION-1 being characterised by the tendency to agglomerate, enhanced cellular uptake and relatively high endothelial toxicity^16^, are not a candidate for further development and potential clinical use. For clinical applications, especially nanoparticle agglomeration may be a decisive factor limiting their use in patients, as it affects both safety and bioavailability.

In contrast to SPION-1, we did not achieve magnetic accumulation of SPION-3 within the experimental conditions tested here. We attribute this to the strong steric repulsion of the surface coating and the smaller iron oxide core size compared to SPION-1 and SPION-2 (Table 1). This could indicate that due to the bulky polymer shell around the particles, the dipole-dipole interactions cannot easily occur in case of SPION-3.

SPION-2, the particle type with good biocompatibility profile^28^, as well as good colloidal and blood stability^29^, were successfully targeted to the specific artery region under external magnetic field gradient. Although the observed peak in the iron content under the tip of the magnet was significantly smaller than for SPION-1, the difference in particle accumulation between magnetically targeted and untargeted samples was very strongly pronounced. Moreover, it is plausible that in physiologic-like situation, where the SPIONs are administered via bolus injection near the target region, the magnetic accumulation of SPION-2 can be further enhanced due to increased probability of dipole-dipole interactions of magnetized SPIONs.

Our present study has several limitations, including the use of cell culture media and diluted SPION suspensions in the flow experiments. The use of cell culture media instead of blood (or a fluid with comparable viscosity) results in the necessity of increasing the flow rate in order to achieve physiologic arterial shear stress levels. Furthermore, in contrast to the bolus administration commonly applied *in vivo*, the use of diluted nanoparticle suspensions decreases the interactions between SPIONs. Both these factors are thus likely to impact the magnetic capture efficacy.

Concerning the model itself, despite careful handling, the inflow and outflow regions (−5, −4 and +4, +5) are prone to a processing damage during the placement of connectors, which may lead to an aberrant accumulation of circulating SPIONs at the injured region (e.g. Fig. 3B). Furthermore, as the connectors are (a) stiff and (b) slightly narrower than the vessel diameter, some recirculation areas at both inflow and outflow zone can occur. Ideally, only the regions from −3 to +3, which experience uniform flow conditions, should be considered in the analyses. In the future, we aim to perform a computational flow dynamics analysis in our model to better estimate the possible effects of local hemodynamic conditions on the SPION capture. Despite the existing limitations, many valuable studies can be devised using this model, including the modifications of arterial wall geometry to mimic the presence of stenosis, or the implantation of stents to investigate the magnetic capture of SPION-loaded cells.

In conclusion, the MDT application using SPION-2 can be envisioned based on the presented results. Collectively, these results also imply that a precise positioning of the external magnet should allow an efficient targeting of certain types of SPIONs to superficial arteries in *vivo*, but more sophisticated magnetic field geometries will be necessary in the case of arteries localised in the deeper body regions. Moreover, as the rheological behaviour of blood cells in the arterial flow may strongly affect the capturing efficacy, further extensive investigations in the presence of whole blood will be needed to better characterise the margination and accumulation of magnetic particles under arterial flow conditions.

## Methods

### Materials

Bovine serum albumin (BSA) and iron (II) chloride tetrahydrate were from Merck (Darmstadt, Germany). Lauric acid, epichlorohydrin, iron (III) chloride hexahydrate and dextran T40 (Mw = 40 kDa) were from Sigma Aldrich (Munich, Germany). NaOH, HCl (25%), NH_3_ (25%), nitric acid (65%w/w), potassium ferrocyanide and agarose were from Roth (Karlsruhe, Germany). All compounds used were of pharmaceutical (Ph. Eur) or highly pure (≥99%) grade and were used without any further purification.

Endothelial cell growth medium was obtained from PromoCell (Heidelberg, Germany) and Ringer solution from Fresenius Kabi (Bad Homburg, Germany). Accutase™ was from PAA Laboratories (Linz, Austria), dispase and nuclear dye Hoechst 33342 were from Life Technologies GmbH (Darmstadt, Germany), annexin V-FITC and DilC_1_ were from Invitrogen (Life Technologies) and propidium iodide (PI) from Sigma. Trichrome stain reagents were purchased from Merck and hematoxylin from Dako (Hamburg, Germany).

### Nanoparticle synthesis and characterisation

The three tested types of SPIONs were synthesized at the Section of Experimental Oncology and Nanomedicine, University Hospital Erlangen.

#### SPION-1

Lauric acid-coated iron oxide nanoparticles were synthesized using a coprecipitation method as described by Tietze *et al*.^2^. Briefly, Fe (II) and Fe (III) salts at a defined molar ratio (Fe^3+^/Fe^2+^ = 3:2) were dissolved in water, followed by addition of NH_3_ solution under stirring. The precipitate was then washed with 1.3% ammonium hydroxide solution, followed by the addition of lauric acid and heating to 90 °C for 4 min under stirring^30^. The resulting lauric acid-coated particles were washed 10 times with 1.3% ammonium hydroxide solution. Prior to their use in cell culture studies, the SPION-1 were stabilised by incubation 1:1 with a freshly prepared 10% BSA solution and sterilised by filtration through a 0.22 μm filter (Roth).

#### SPION-2

Lauric acid/BSA-coated iron oxide nanoparticles were synthesized by co-precipitation, subsequent *in situ* coating with lauric acid, and formation of an artificial albumin corona as described by Zaloga *et al*.^29^. Briefly, Fe (II) and Fe (III) salts at a defined molar ratio (Fe^3+^/Fe^2+^ = 2) were dissolved in 20 mL of water and stirred at 80 °C under argon atmosphere, followed by addition of 20 mL of NH_3_ solution (25%). The solution was heated to 90 °C and 1.25 g lauric acid, dissolved in acetone, was added. The brownish suspension was left to homogenate for 30 min at 90 °C. The suspension was then dialysed multiple times against ultrapure water. Subsequently, SPIONs were stabilised by incubation with a freshly prepared 20% BSA solution, purified by centrifugal ultrafiltration (molecular weight cut-off 100 kDa), and sterilized by filtration through a 0.22 μm filter.

#### SPION-3

For the preparation of dextran-coated iron oxide nanoparticles, the synthesis method described by Unterweger *et al*. was used^31^. Briefly, Fe (II) and Fe (III) salts in molar ratios (Fe^3+^/Fe^2+^ = 2) as well as 1.75 g of dextran T40 were dissolved in water. After cooling to 4 °C under continuous stirring and argon atmosphere, 5 mL of ice-cold 25% NH_3_ was added. After 5 min, the reaction mixture was heated and kept at 75 °C for a further 40 min, followed by cooling to RT and dialysis (molecular weight cut-off 8 kDa). The mixture was then cleared from excess dextran and concentrated using ultrafiltration (molecular weight cut-off 100 kDa). To stabilise the dextran coating, crosslinking was performed by adding 4 mL of epichlorohydrine dropwise to the nanoparticle suspension after alkalization with NaOH under vigorous stirring for 24 h. The solution was then dialysed against water, concentrated by ultrafiltration and sterile filtered through 0.22 μm membrane.

The extensive physicochemical characterisation of these particles was reported in the previous publications^28^,^29^,^31^,^32^,^33^ and is briefly summarised in the Table 1.

### Umbilical artery preparation and characterisation

Human umbilical arteries were isolated from freshly collected umbilical cords (kindly provided by the Dept. of Gynaecology, Prof. Beckmann, University Hospital Erlangen). The study was conducted according to the Declaration of Helsinki and the relevant national guidelines. The use of human material was approved by the local ethics committee at the University Hospital Erlangen (review No. 4449). Written informed consent was obtained from donors.

Immediately after the birth, umbilical cords were placed in glass containers containing saline supplemented with antibiotics (1% penicillin/streptomycin) and fungicide patricin. Within 1–4 days post-partum, the cords were used for isolation of primary HUVECs from umbilical vein. After HUVECs isolation was completed, the umbilical arteries were prepared from the cords, washed and cut in 13 cm fragments, used for the flow experiment as described in detail below.

To characterise the arterial specimens, 5 μm-thick paraffin sections were prepared and vessel morphology was analysed using Crossman’s trichrome stain for muscle and collagen, whereby the collagen fibres were stained green, the nuclei were stained black and the cytoplasm and muscle tissue were stained red (see Supplementary methods).

To characterise the HUAECs phenotype, a dedicated fragment of the artery was filled with dispase solution (activity of 2.4 U/mL). After 30 min incubation, umbilical artery was delicately massaged to detach the cells. The solution containing HUAECs was collected in Falcon tubes, followed by dispase inactivation with endothelial cell medium containing 10% foetal calf serum. After centrifugation, HUAECs were seeded in the culture flasks and grown until confluence in supplemented endothelial cell growth medium, at humidified 5% CO_2_ atmosphere. Following the harvesting using Accutase^TM^ and centrifugation, cells were stained with annexinV-FITC to detect apoptotic cells, PI to identify necrotic cells, Hoechst 33342 and DilC_1_ to estimate the mitochondrial membrane potential. Fluorescence was measured with flow cytometer (Gallios, Beckman Coulter, Fullerton, USA). Electronic compensation was used to eliminate bleed through fluorescence.

### Flow experiments

Plastic Luer connectors (Novodirect, Kehl, Germany), were fixed at each end of a 13 cm long fragment of previously isolated umbilical artery with surgical thread to facilitate rinsing and perfusion. Fragments were then rinsed multiple times with Ringer solution to remove the remaining blood clots and placed in a plastic container (13 × 7 × 1.5 cm) as shown in Fig. 1. The artery was subsequently filled with endothelial cell culture medium (preheated to 37 °C) to prevent the lumen collapse and embedded in agarose gel, cooled to 40 °C to avoid tissue damage. A closed circuit connected to the peristaltic pump (model ISM 915, Ismatec, Wertheim, Germany) and SPION reservoir (Fig. 1) via Ismaprene tubes (Ismatec) was used to perfuse the arteries with medium containing SPIONs (30 μg/mL) with or without an external magnetic force for 30 min, at the flow rate of 4.8 mL/min (corresponding to the shear stress of 5 dyne/cm^2^). In the experiments with magnetic field gradient (Siemens electromagnet, Siemens AG Erlangen, Germany^34^), the magnetic field gradient at the tip of the pole shoe was set for 72 T/m, which corresponded to about 40 T/m field gradient at the centre of artery lumen. In some experiments, the capture efficacy was investigated under reduced currents, which corresponded to about 30 T/m at the centre of artery lumen. Unless stated otherwise, the tip of the magnet was positioned directly on top of the agarose gel surface, in a 5 mm distance from the wall of embedded artery. As controls, SPIONs without external magnetic field gradient or medium only were used. After the experiment, the arteries were rinsed with Ringer solution for 2 min. Subsequently, the models were disconnected from the pump and arteries were cut into 11 segments of 1 cm length, numbered (−5, …, 0, …, +5) from in-flow to out-flow as indicated (Fig. 1B), followed by additional rinsing of single sections with Ringer solution. For each SPION type and each experimental condition, 5 repetitions were performed for subsequent iron content measurement and 3 for histological evaluation.

### Iron content measurement with AES

The tissue iron concentration was quantified with microwave plasma atomic emission spectroscopy (MP-AES, 4200 device, Agilent). Following the flow experiment, the rinsed tissue segments were dried for 2 h at 90 °C, dissolved in 100 μL of 65% nitric acid for 10 min at 95 °C and 600 rpm using a thermomixer. After addition of 900 μL of water, the emission spectrum of the samples was analysed and compared to the standard curves. Tissue iron concentration values were given as ng Fe per artery segment.

### Tissue iron content by histology

The tissue segments were stained with Prussian blue to assess the uptake of circulating SPIONs. Briefly, the artery segments were placed in embedding cassettes, fixed in 4% formaldehyde solution (Roth) in PBS buffer for 2 days. Afterwards, samples were dehydrated in an ascending isopropanol sequence and finally embedded in paraffin. Blocks were then cut using the microtome and the paraffin-embedded serial sections of 4-μm-thickness were dewaxed in xylene, rehydrated in ethanol, and immersed in 1:1 solution of hydrochloric acid (2%) and potassium ferrocyanide (2%) for 30 min at RT. Nuclei of the cells were counterstained with Fast Red, followed by rinsing with distilled water. As a mounting medium, Mowiol (Sigma-Aldrich) was added to extend staining durability. Images were taken using Axio Observer.Z1 microscope (Zeiss, Jena, Germany).

### Statistics

The differences in magnetic accumulation of SPIONs between the different segments of the same artery model were calculated after performing a normality test (Shapiro-Wilk), using one way ANOVA with Holm-Sidak pairwise comparisons, or one way ANOVA on Ranks with Tukey post-hoc test. The comparisons between the same segments but different experimental conditions were done using t-test in samples that passed the normality test and non-parametric Mann-Whitney U-test in those, which failed the normality test. Data were expressed as mean ± SEM, unless stated otherwise. P < 0.05 was considered statistically significant.

## Additional Information

**How to cite this article**: Janikowska, A. *et al*. A novel human artery model to assess the magnetic accumulation of SPIONs under flow conditions. *Sci. Rep.* **7**, 42314; doi: 10.1038/srep42314 (2017).

**Publisher's note:** Springer Nature remains neutral with regard to jurisdictional claims in published maps and institutional affiliations.

## Supplementary Material

Supplementary Data

## Figures and Tables

**Table 1:** Physicochemical characterisation of the tested SPIONs.

	SPION-1	SPION-2	SPION-3
Core size (TEM) [nm]	15.3 ± 3.6	8.9 ± 2.2	4.3 ± 0.9
Hydrodynamic diameter [nm]	126	79	78
ζ-potential [mV]	−34.6	−37.3	+0.1

The hydrodynamic size (Z-average size) and ζ-potential of the nanoparticles were determined with a Zetasizer Nano ZS (Malvern). The average core size was measured on transmission electron microscopy (TEM) images.
