# Supplementary material for: A novel human artery model to assess the magnetic accumulation of SPIONs under flow conditions
Source: Sci Rep. 2017 Feb 8;7:42314. doi: 10.1038/srep42314 (PMC5296745; doi:10.1038/srep42314)
Supplement: Supplementary Data [file srep42314-s1.doc]

**Online supplement**

**SREP-16-40475A**

**A novel human artery model to assess the magnetic accumulation of SPIONs under flow conditions**

Agata Janikowska, Jasmin Matuszak, Stefan Lyer, Eveline Schreiber, Harald Unterweger, Jan Zaloga, Jürgen Groll, Christoph Alexiou and Iwona Cicha.

**Supplementary Methods**

***Crossman’s trichrome staining***

To characterise the arterial specimens, 5 µm-thick paraffin sections were prepared and vessel morphology was analysed using Crossman’s trichrome stain for muscle and collagen. Briefly, samples were stained with hematoxylin (Dako) for 10 minutes, followed by rinsing with tap water for 10 minutes. Afterwards, the samples are stained for approximately 1 minute with a mixture of acid fuchsine (Merck) and acridine orange (Merck) and subsequently washed with distilled water. The resulting red staining was differentiated with 1% molybdophosphoric acid until the connective tissue was decolorized. After washing with distilled water, staining with light green (Merck) was performed for 5 min with subsequent washing. Following dehydration in isopropanol gradient and xylene, samples were embedded using a durable medium (Roti-Histo Kit, Roth).

***HUAECs characterisation***

To characterise the HUAECs phenotype, a dedicated fragment of the artery was filled with dispase solution (activity of 2.4 U/mL). After 30 min incubation, umbilical artery was delicately massaged to detach the cells. The solution containing HUAECs was collected in Falcon tubes, followed by dispase inactivation with endothelial cell medium containing 10% foetal calf serum. After centrifugation, HUAECs were seeded in the culture flasks and grown until confluence in medium with endothelial cell growth supplement containing 5% foetal calf serum, 4 μL/mL heparin, 10 ng/mL epidermal growth factor, 1 μg/mL hydrocortisone, 50 μg/mL gentamycin sulphate, and 50 ng/mL amphotericin B, at humidified 5% CO_2_ atmosphere.

Following the harvesting using Accutase^TM^ and centrifugation, cells were stained with annexin V-FITC (AxV-FITC) to detect apoptotic cells, propidium iodide (PI) to identify necrotic cells, Hoechst 33342 and DilC_1_ to estimate the mitochondrial membrane potential. Fluorescence was measured with flow cytometer (Gallios, Beckman Coulter, Fullerton, USA). Electronic compensation was used to eliminate bleed through fluorescence.

**Supplementary Results**

***Artery characterisation by histology***

The umbilical arteries used in this study had diameter of 2.15±0.09 mm (mean ± SEM; median: 2.11 mm; range 1.32-2.98 mm) as measured in n=18 histological specimens. The arteries were isolated from the umbilical cords between day 1 and day 4 post-partum. Only the intact artery fragments were used in order to avoid the leakage of SPIONs and unspecific accumulation in the artery wall. The example images of vessel morphology analysed by Crossman’s trichrome staining are shown in **Suppl. Fig. 1**.

***HUAECs characterisation***

The morphology of HUAECs isolated from several different umbilical arteries was analysed using IncuCyte FLR (live-cell imaging microscope, EssenBioscience). The example images of sub-confluent and confluent HUAECs are shown in the **Suppl. Fig. 2**. As controls, the images of HUVECs at similar confluence are shown. There were no morphological differences between the isolated HUAECs and the routinely cultured HUVECs.

**
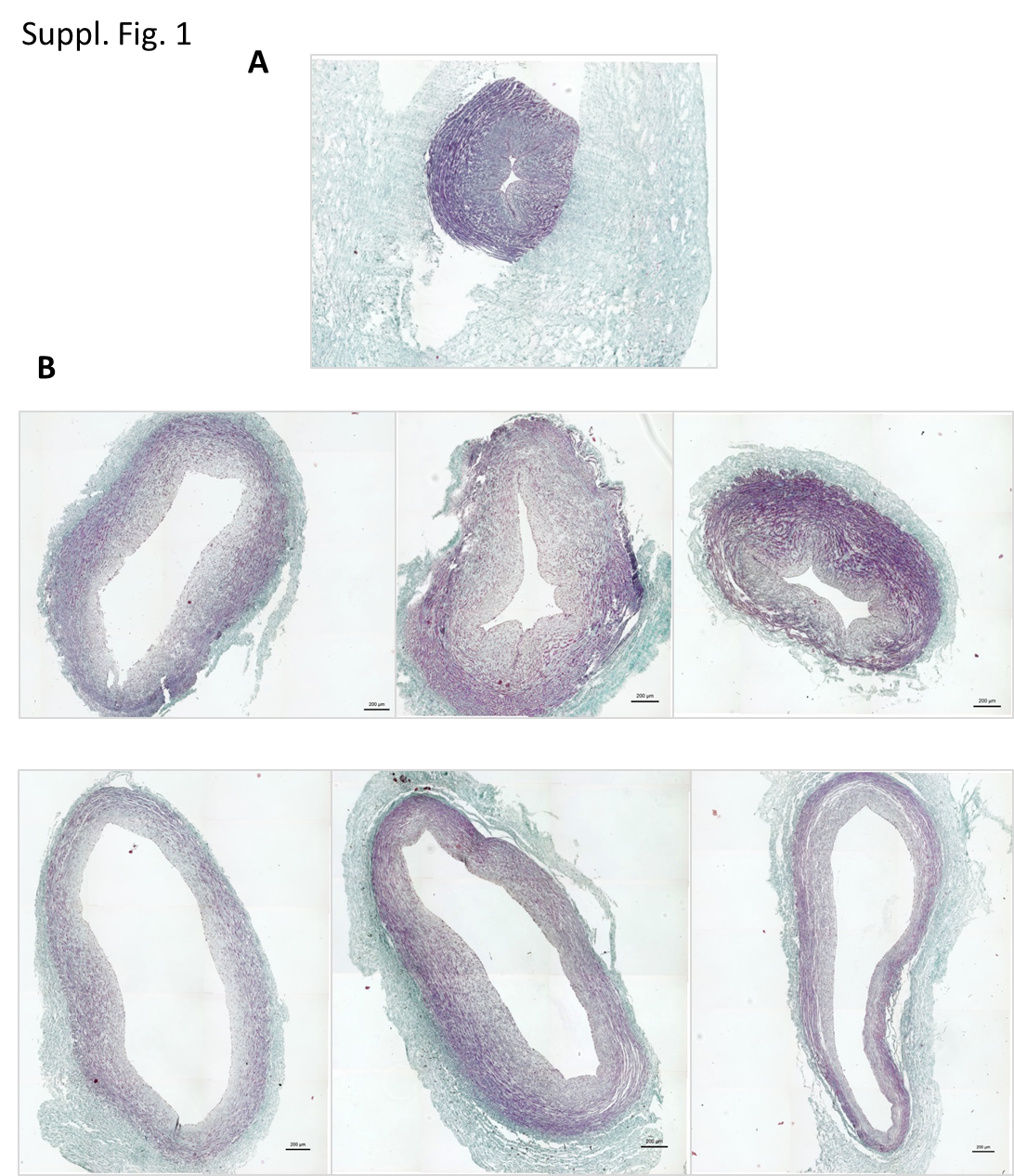
**

**Suppl. Figure 1. Histological characterisation of umbilical arteries.** Representative images of different artery segments stained with Crossman’s trichrome stain for muscle and connective tissue. (A) Artery section surrounded with the native umbilical cord tissue. (B) Example artery sections showing the typical arterial wall morphology with high content of smooth muscle cells. Note the large inter-donor variability in the lumen diameter and thickness of tunica media. Scale bar: 200 µm.


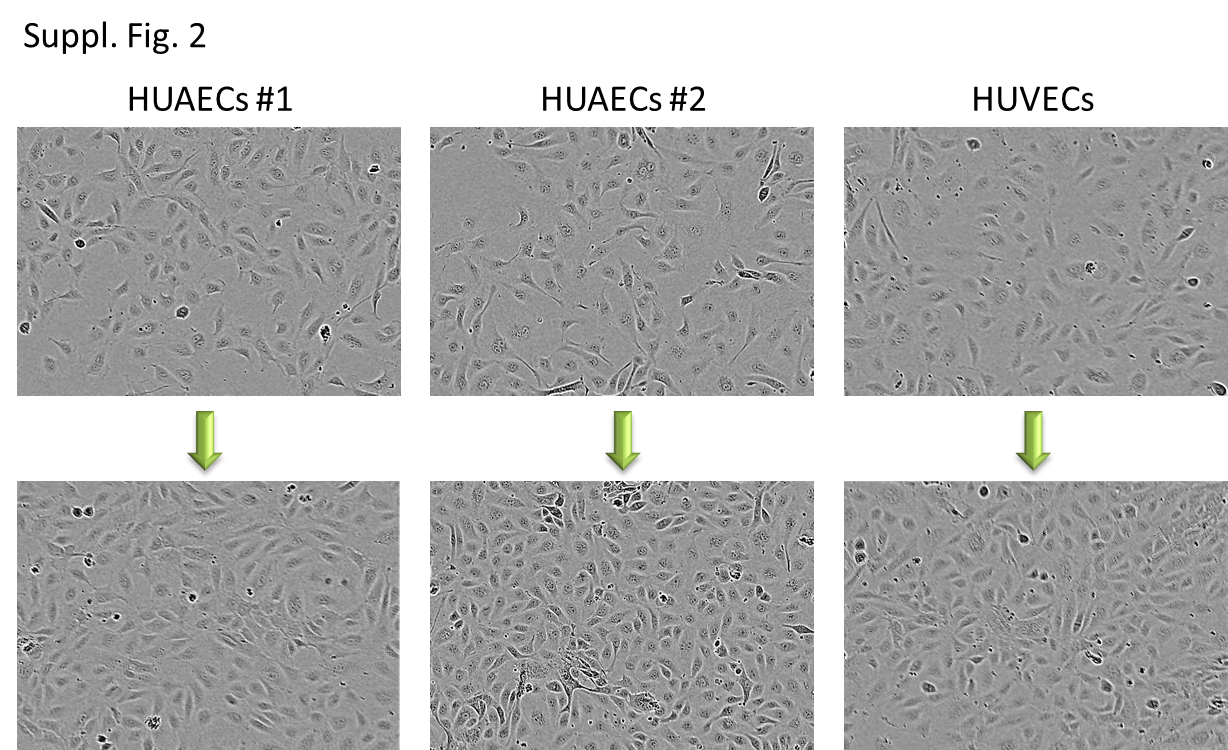


**Suppl. Figure 2. Morphology of human umbilical artery endothelial cells (HUAECs).** Representative images of 2 different populations of HUAECs are shown at increasing confluence. Example images of routinely isolated HUVECs are shown for comparison. The ECs from the different umbilical vascular beds do not differ in size or morphology. Images were taken with live-cell imaging microscope (IncuCyte FLR) at 10x objective magnification.

AxV/PI stainnig was used to determine cell viability status of the isolated HUAECs cultured for 7 days. AnnexinV binds to phosphatidylserine (PS), which is located on the intracellular side of the plasma membrane in healthy cells. During apoptosis, PS molecules flip to the extracellular side of the membrane and are detected by flow-cytometry. PI is a membrane-impermeable stain. During necrosis, which is characterized by ruptured plasma membrane, PI can permeate and intercalate into the DNA. DilC_1_/Hoechst staining was used to estimate the mitochondrial membrane potential, which is reduced in dying or dead cells. Hoechst stains the DNA and marks healthy cells, whereas DilC_1_ accumulates in active mitochondria. As shown in **Suppl. Fig. 3**, there were no major differences between HUAECs and HUVECs viability in culture, about 79% of HUAECs and 80% of HUVECs being negative for both PI and AnnexinV. According to DilC_1_/Hoechst staining, above 75% of HUAECs were viable.

**
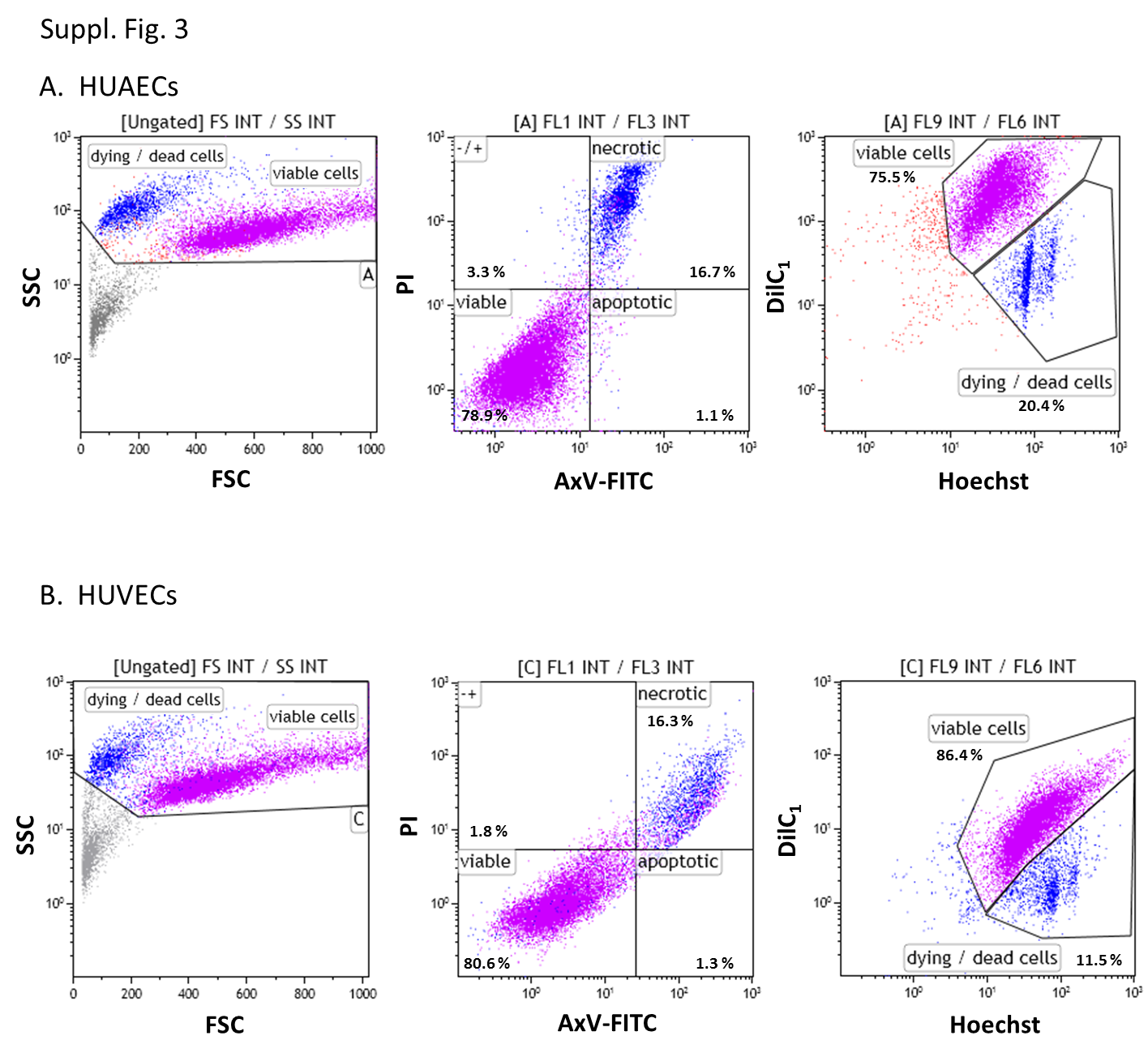
**

**Suppl. Figure 3. Comparative flow-cytometric analysis of (A) HUAECs and (B) HUVECs.** Left panel: Side-scatter and forward-scatter intensities. Middle panel: AnnexinV/FITC and PI staining. Right panel: DilC_1_/Hoechst staining. The percentages of viable, apoptotic and necrotic cells are indicated.
